# Interobserver variability in clinical target volume delineation in anal squamous cell carcinoma

**DOI:** 10.1038/s41598-021-82541-5

**Published:** 2021-02-02

**Authors:** Kyung Su Kim, Kwang-Ho Cheong, Kyubo Kim, Taeryool Koo, Hyeon Kang Koh, Ji Hyun Chang, Ah Ram Chang, Hae Jin Park

**Affiliations:** 1 Department of Radiation Oncology, Dongnam Institute of Radiological and Medical Sciences, Busan, Republic of Korea; 2 Department of Radiation Oncology, Ewha Womans University College of Medicine, 1071 Anyangcheon-ro Yangcheon-gu, Seoul, 07985 Republic of Korea; 3 Department of Radiation Oncology, Hallym University Sacred Heart Hospital, Anyang, Republic of Korea; 4 Department of Radiation Oncology, Konkuk University School of Medicine, Seoul, Republic of Korea; 5 Department of Radiation Oncology, Seoul Metropolitan Government, Seoul National University Boramae Medical Center, Seoul, Republic of Korea; 6 Department of Radiation Oncology, Soonchunhyang University Seoul Hospital, Soonchunhyang University College of Medicine, Seoul, Republic of Korea; 7 Department of Radiation Oncology, Hanyang University College of Medicine, 222-1 Wangsimni-ro, Seongdong-gu, Seoul, 04763 Republic of Korea

**Keywords:** Cancer, Oncology

## Abstract

We evaluated the inter-physician variability in the target contouring of the radiotherapy for anal squamous cell carcinoma (ASCC). Clinical target volume (CTV) of three patients diagnosed with ASCC was delineated by seven experienced radiation oncologists from multi-institution. These patients were staged as pT1N1a, cT2N0, and cT4N1a, respectively, according to 8^th^ edition of the American Joint Committee on Cancer staging system. Expert agreement was quantified using an expectation maximization algorithm for Simultaneous Truth and Performance Level Estimation (STAPLE). The maximum distance from the boundaries of the STAPLE generated volume with confidence level of 80% to those of the contour of each CTV in 6 directions was compared. CTV of pelvis which includes primary tumor, perirectal tissue and internal/external iliac lymph node (LN) area (CTV-pelvis) and CTV of inguinal area (CTV-inguinal) were obtained from the seven radiation oncologists. One radiation oncologist did not contain inguinal LN area in the treatment target volume of patient 2 (cT2N0 stage). CTV-inguinal displayed moderate agreement for each patient (overall kappa 0.58, 0.54 and 0.6, respectively), whereas CTV-pelvis showed substantial agreement (overall kappa 0.66, 0.68 and 0.64, respectively). Largest variation among each contour was shown in the inferior margin of the CTV-inguinal. For CTV-pelvis, anterior and superior margin showed the biggest variation. Overall, moderate to substantial agreement was shown for CTV delineation. However, large variations in the anterior and cranial boarder of the CTV-pelvis and the caudal margin of the CTV-inguinal suggest that further studies are needed to establish a clearer target volume delineation guideline.

## Introduction

Anal squamous cell carcinoma (ASCC) is a rare type of cancer comprising only 0.3% of all malignancy^1^. Concurrent chemoradiation (CCRT) with 5-fluorouracil plus mitomycin-C has been the standard therapy for ASCC^2^. Radiotherapy volume encompasses primary tumor within mesorectum and elective nodal area including obturator, internal iliac, external iliac, presacral and/or inguinal lymph node (LN) area. Advances in radiation therapy technology have led IMRT to replace 3-dimensional (3D) conformal radiotherapy. Whereas large randomized trials utilized mainly 3D conformal radiotherapy^3,4^, recent clinical trials are testing the application of intensity modulated radiotherapy (IMRT) to reduce toxicity by minimizing radiation dose to normal organs without compromising target coverage^5,6^. Accurate and well-defined target volume definition is the essential element of IMRT to avoid missing the treatment target and to minimize the dose to surrounding normal tissue. Accurate target delineation is even more critical in the era of image guided radiotherapy (IGRT) using daily conebeam CT or ultrasonogram, because IGRT sided by incorrect target volumes only allows to precisely hit the wrong ones^7^. Moreover, it is also crucial to reduce toxicity profile of CCRT by specifically targeting organs at risk, such as bowel for gastro-intestinal (GI), genitals for genito-urinary (GU) and bone marrow for hematologic toxicity^8–10^.

There existed different contouring guidelines for IMRT of ASCC^11–13^. While these guidelines provide robust evidence and reproducibility in routine radiation therapy at the clinic, there are still ambiguous definition in the field margin of the clinical target volume (CTV) for elective LN irradiation. Moreover, there may be inter-physician variation resulting from differences in experience, and/or various institutional policy. To date, no studies have shown how varying CTV’s are among experienced radiation oncologists in the real-world clinical settings.

Therefore, in the current study, we evaluated the inter-physician variability of target contouring of the radiotherapy for ASCC.

## Materials and methods

Three ASCC patients previously treated with radiotherapy were selected in this study. All patients were diagnosed as ASCC through pathologic examination. For staging work up, all patients underwent colonoscopy, abdomino-pelvic computed tomography (CT), magnetic resonance image (MRI) of pelvis, and positron emission tomography (PET)/CT. TNM staging was described according to the 8th edition of the American Joint Committee on Cancer staging system. Patient 1 was a 49-year-old woman diagnosed with stage pT1N1a ASCC. The patient underwent excisional biopsy. Pathologic examination revealed a 1.4 cm tumor and the resection margin was positive. There was a metastatic LN (1.2 cm in size) in the left inguinal chain which was identified in CT and MRI, and increased maximum standardized uptake value (SUVmax) of 5.0 was observed in PET/CT images. Patient 2 was a 77-year-old woman with stage cT2N0. The tumor size was 3.9 cm. No LN was identified in the CT, MR and PET/CT images. Patient 3 was a 72-year-old woman with stage cT4N1a. About 4.5 cm-sized tumor involved anus, perineum and posterior wall of vagina. There was a 1.2 cm-sized and pathologically confirmed LN in the right inguinal area, which showed increased SUVmax value of 3.07 in PET/CT images. Clinical information of the patients was described in Table 1.

**Table 1:** Patient characteristics.

Patient no.	Sex/age	Stage	Surgery	T stage	N stage	Target delineation
1	F/49	pT1N1a	Excisional biopsy	1.2 cm margin positive	1.2 cm-sized, PET-positive LN in left inguinal area	CTV-pelvis CTV-inguinal
2	F/77	cT2N0	None	3.9 cm sized	Node negative	CTV-pelvis CTV-inguinal
3	F/72	cT4N1a	None	4.5 cm sized invading vagina	1.2 cm-sized, PET-positive lymph node in right inguinal area. Pathologically confirmed by needle biopsy	CTV-pelvis CTV-inguinal

After approval of institutional review board (IRB) of Dongnam Institute of Radiological and Medical Sciences(DIRAMS) (IRB no. D-1809-035-002), clinical information including medical history, colonoscopy, abdomino-pelvic CT, pelvic MRI and PET/CT images and pathologic report of these patients were sent to seven radiation oncologists practicing in different institutions. Participants’ written informed consent was waived by the IRB of DIRAMS since the data was provided as de-identified form. Our research was performed in accordance with relevant guidelines and regulations. The careers of these clinicians range from three to 13 years. They were asked to delineate CTV of pelvis (CTV-pelvis), which includes primary tumor, perirectal tissue, and presacral/obturator/internal/external iliac LN area. Delineation of CTV for inguinal LN area (CTV-inguinal) was decided to the clinician’s decision.

After acquisition of Dicom file of contours from each institution, we analyzed the target volume using MATLAB software (MathWorks, Natick, MA). For the quantification of the agreement in volume definition, we analyzed using two different method. Generalized conformity index (CI_gen_) can be simplified into an expression as:${\mathrm{CI}}_{gen}=\frac{{\sum }_{pairs i,j}\left|{A}_{i}\cap {A}_{j}\right|}{{\sum }_{pairs i,j}\left|{A}_{i}\cup {A}_{j}\right|}$ , where A_i_ and A_j_ represent the volumes described by the i-th and j-th physicians, respectively. Here, CI_gen_ < 0.5 is generally considered a weak correlation, while CI_gen_ ≥ 0.7 is acceptable^14^. For the other analysis of the agreement among CTVs of different physicians, we applied the Simultaneous Truth and Performance Level Estimation (STAPLE) algorithm included in the Computational Environment for Radiotherapy Research (CERR) software. This is known as the expectation–maximization algorithm^15^; it estimates the true contour by implementing an optimization process through the spatial uniformity condition by weighting the performance level of each delineated contour. The performance level is the probability of how each contour is close to the virtual true contour^16^. In CERR, sensitivity, specificity, and agreement level measurements are expressly provided as an apparent agreement, a kappa-corrected agreement, and a STAPLE-estimated probability. The apparent agreement evaluates the probability of correspondence between observers for each voxel. The kappa-corrected agreement is the corrected consistency to exclude the possibility of coincidence^15^. In general, a kappa value of < 0.00 indicates poor agreement; 0.00–0.20, slight agreement; 0.21–0.40, fair agreement; 0.41–0.60, moderate agreement; 0.61–0.80, substantial agreement; and 0.81–1.00, almost perfect agreement^17^. Based on the STAPLE analysis, we generated a contour for each CTV set using the 80% confidence level and used it as a reference (CTV-80) for comparison with each CTV.

For the difference analysis, we calculated the maximum distance from the boundary of the CTV-80 to that of each CTV contour in six directions. This distance does not necessarily have to be on the same plane along the axis. Pirateplot^18^ of these values, showing descriptive statics (mean and median) and inferential statistics (95% confidence interval) was generated using R software version 3.6 (R Core Team, 2020. R: A language and environment for statistical computing. R Foundation for Statistical Computing, Vienna, Austria. https://www.R-project.org/).

## Results

**Figure 1:**
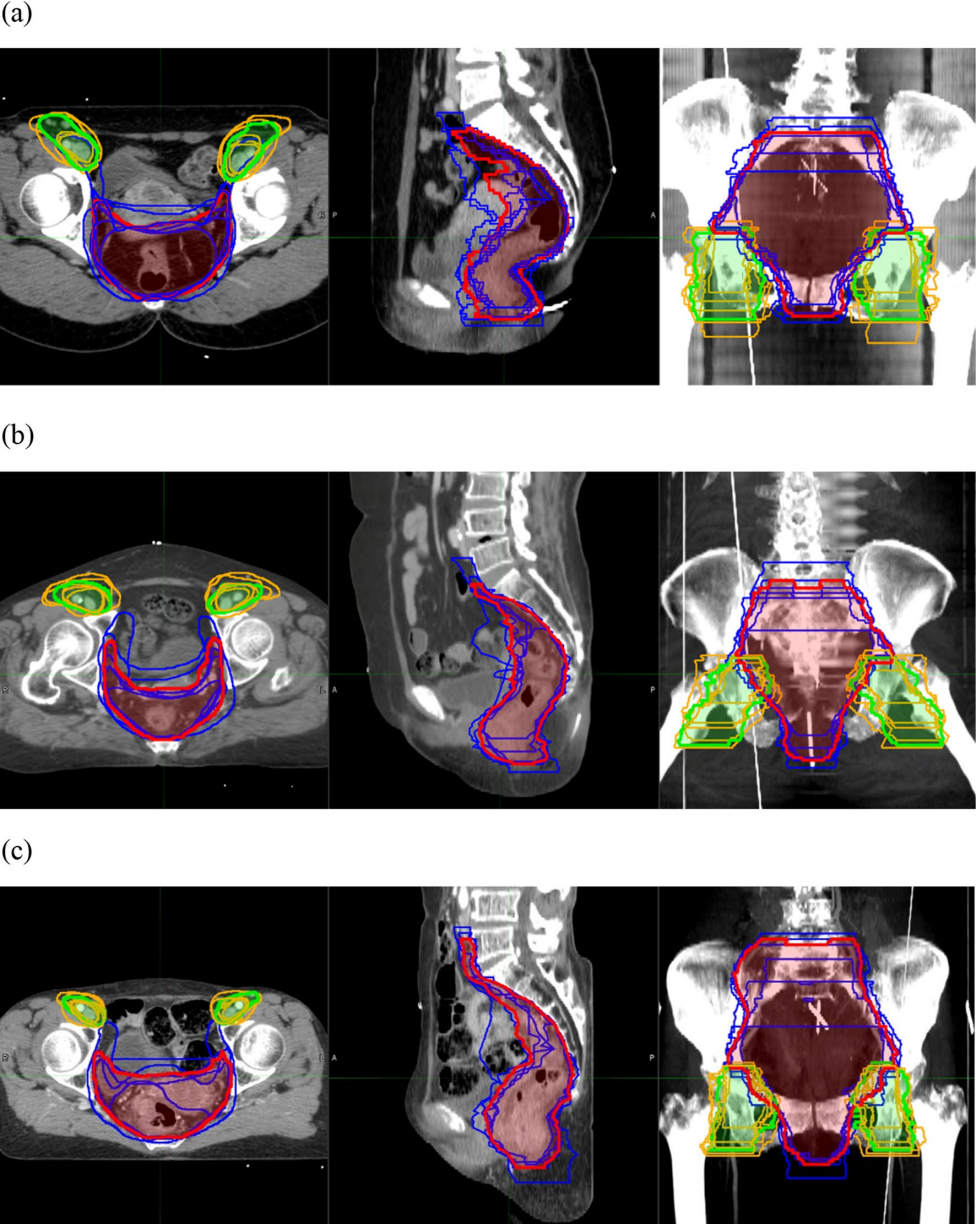
CTV-pelvis (dark blue) and CTV-inguinal (orange) of 7 radiation oncologists and STAPLE generated contour of CTV-pelvis (red) and CTV-inguinal (green) with confidence level of 80%. **(a)** Patient 1; **(b)** Patient 2; **(c)** Patient 3. Figures were generated using MIM 6.9.7 (MIMVista Corp, Cleveland, Ohio).

**Figure 2:**
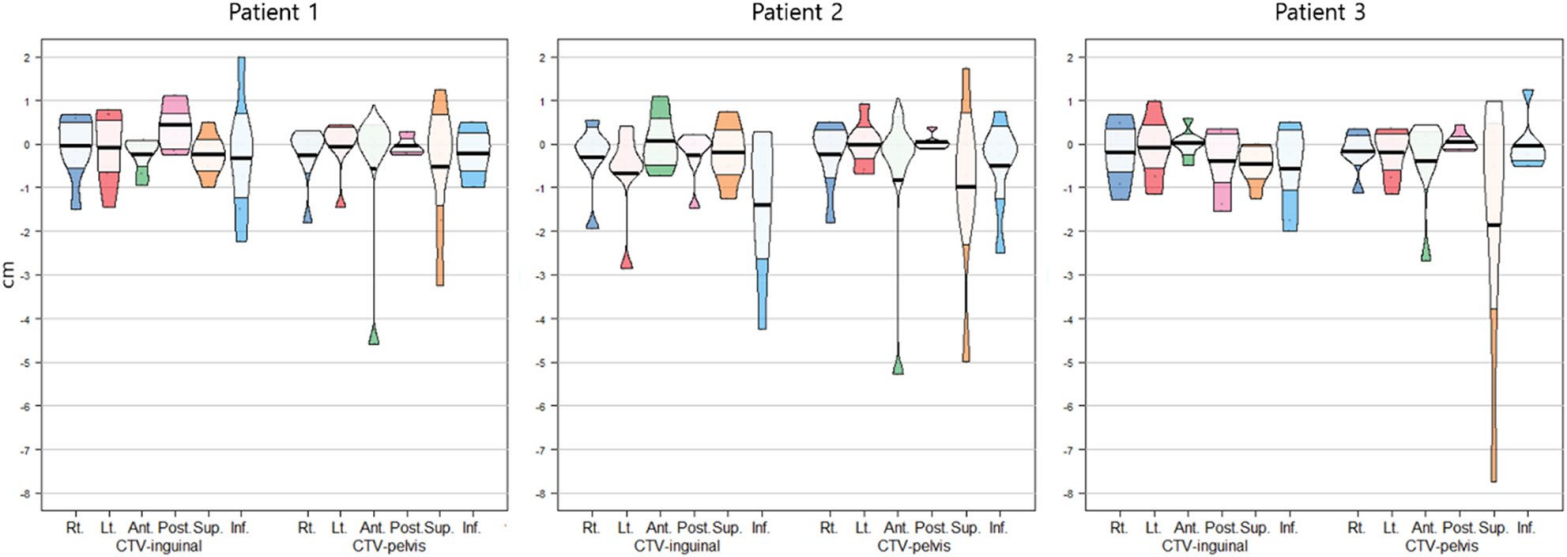
Pirateplot of differences of borders in 6 directions between each CTV and STAPLE generated CTV with confidence level of 80% (CTV-80). Positive values represent that border of each CTV is larger than CTV-80 in each direction. Plots were generated using R software version 3.6 (R Core Team, 2020. R: A language and environment for statistical computing. R Foundation for Statistical Computing, Vienna, Austria. https://www.R-project.org/).

Each CTVs was obtained from the seven radiation oncologists. One radiation oncologist did not contain inguinal LN area in the treatment target volume of patient 2. Analysis of CTV-inguinal of patient 2 was conducted using six contours. Volume and level of agreement of the contours was described in Table 2. Mean and standard deviation of CTV-inguinal was 181.37 ± 65.33 cm^3^, 158.04 ± 65.37 cm^3^ and 91.69 ± 335.59 cm^3^ for each patients, and those of CTV-pelvis were 633.30 ± 158.98 cm^3^, 658.32 ± 140.16 cm^3^ and 609.24 ± 143.21 cm^3^, respectively. Overall kappa value ranged from 0.54 to 0.75. CTV-inguinal displayed moderated agreement for each patient, whereas CTV-pelvis showed substantial agreement. The CI_gen_ value ranged from 0.45 to 0.55. CI_gen_ of CTV-pelvis of three patients had values over 0.5, whereas CI_gen_ of CTV-inguinal was below 0.5. Each CTV of seven clinicians and CTV-80 were delineated in Fig. 1. The differences of boarders between CTV-80 and each CTV along the 6 directions were described in Table 3 and Fig. 2. For CTV-inguinal, the largest variation among each contour was shown in the inferior margin. For CTV-pelvis, anterior and superior margin showed the biggest variation. (Fig. 2).

**Table 2:** Summary of clinical target volume (CTV) statistics.

Parameters	Patient 1		Patient 2		Patient 3	
	CTV-inguinal	CTV-pelvis	CTV-inguinal	CTV-pelvis	CTV-inguinal	CTV-pelvis
Volume minimum (cm^3^)	65.08	409.89	107.69	406.65	34.66	435.95
Volume maximum (cm^3^)	264.72	947.81	284.87	918.32	151.74	879.63
Volume mean (cm^3^)	181.37	633.30	185.04	658.32	91.69	609.24
SD (cm^3^)	65.33	158.98	65.37	140.16	35.59	143.21
Volume union (cm^3^)	369.27	1129.17	386.54	1195.15	185.08	1150.10
Volume intersection (cm^3^)	50.65	250.16	40.86	262.05	28.31	215.00
Volume of STAPLE generated contour with confidence level of 80%	212.91	716.78	226.53	730.22	105.95	688.08
Overall kappa	0.58	0.66	0.54	0.68	0.60	0.64
Mean sensitivity	0.69	0.76	0.65	0.77	0.67	0.75
SD of sensitivity	0.24	0.14	0.23	0.13	0.23	0.14
Mean specificity	0.98	0.98	0.98	0.98	0.99	0.98
SD of specificity	0.01	0.02	0.01	0.02	0.02	0.02
p-value	0.00	0.00	0.00	0.00	0.00	0.00
CI_gen_	0.48	0.55	0.45	0.53	0.49	0.52

*SD* standard deviation, *CI*_*gen*_ generalized conformity index.

**Table 3:** Difference of borders along the 6 direction between each clinical target volume (CTV) and STAPLE generated CTV with confidence level of 80% (CTV-80).

Patient	CTV	Right	Left	Anterior	Posterior	Superior	Inferior
1	Inguinal	− 0.05 ± 0.76	− 0.08 ± 0.85	− 0.24 ± 0.41	0.43 ± 0.55	− 0.25 ± 0.48	− 0.32 ± 1.37
1	Pelvis	− 0.26 ± 0.72	− 0.06 ± 0.65	− 0.57 ± 1.79	− 0.04 ± 0.22	− 0.54 ± 1.5	− 0.21 ± 0.62
2	Inguinal	− 0.31 ± 0.86	− 0.68 ± 1.13	0.07 ± 0.67	− 0.27 ± 0.62	− 0.21 ± 0.7	− 1.42 ± 1.70
2	Pelvis	− 0.25 ± 0.8	− 0.01 ± 0.54	− 0.83 ± 2.01	0.03 ± 0.17	− 1.0 ± 2.09	− 0.5 ± 1.06
3	Inguinal	− 0.2 ± 0.72	− 0.08 ± 0.72	0.01 ± 0.33	− 0.4 ± 0.78	− 0.46 ± 0.44	− 0.57 ± 0.98
3	Pelvis	− 0.17 ± 0.48	− 0.21 ± 0.57	− 0.41 ± 1.05	0.04 ± 0.21	− 1.86 ± 2.99	− 0.04 ± 0.60

Number represented mean ± standard deviation in centimeter.

Positive values represents that border of each CTV is larger than CTV-80 in each direction.

## Discussion

The result of the current study demonstrated variations among radiation oncologists in the CTV delineation of ASCC. Overall kappa and CI_gen_ values demonstrated that CTV-inguinal had less agreement level than CTV-pelvis among the physicians.

There are three known contouring guidelines for IMRT of ASCC. These are Radiation Therapy Oncology Group (RTOG) consensus guideline^11^, Australasian Gastrointestinal Trials Group (AGITG) guideline^13^ and British National Guidance (BNG)^12^. In all these guidelines, the superior border of the CTV-pelvis is recommended as the bifurcation of the common iliac artery into the external and internal iliac arteries. However, in the current study, cranial border of the CTV-pelvis showed biggest variation (Fig. 2). The variation of the anterior and cranial border of the CTV-pelvis would be due to the concerning about the common iliac LN metastasis. Dapper et al. compared the three existing guidelines and suggested optimal CTVs considering the PET imaging-based LN distribution of 22 ASCC patients^19^. In three patients with extensive nodal involvement, there were LNs located superiorly to the recommended border of CTV-pelvis. However, common iliac LN relapse was uncommon in the ASCC. In the study reporting the pattern of relapse after definitive CCRT for ASCC using IMRT, common iliac LN relapse accounted for only 4.1 percent^20^. Moreover, Tomasoa et al. did not report any recurrence above the level of S3^21^. Using the PET/CT evaluation for the LN involvement before treatment, bifurcation of the internal/internal iliac arteries would be sufficient as a cranial border for the CTV of pelvic LN.

Regarding the inguinal region, there was a discrepancy in the defining CTV. The anatomy in the inguinal region is very complex due to large differences between the individuals. Therefore, the three guidelines have different recommendations for inferior inguinal margins. The RTOG defines the caudal margin “2 cm caudal to the saphenous/femoral junction”, the BNG determines the “lesser trochanter” and the AGITG identifies “the lower edge of the ischial tuberosities” as a compromise between saphenous/femoral junction and sartorius/adductor longus junction^22^. Dapper et al. also pointed out that 10% to 29% of the inguinal LN was not covered by the CTV’s of RTOG, AGITG and BNG guidelines^19^. Twenty percent of the inguinal LN was located inferiorly to the RTOG inguinal CTV and only four LN’s were located below the lower edge of the ischial tuberosity, suggesting that inferior border of the inguinal LN should be anal verge or 2 cm caudal to anal verge if extensive disease or multiple LN’s^19^. Because of the large anatomical variation of the inguinal area, more clear definition of the target volume based on the obvious anatomical landmarks is needed for the consistent target contouring among physicians.

Regarding elective inguinal irradiation, one radiation oncologist did not include inguinal radiotherapy in patient 2 with cT2N0 stage ASCC in the current study. There is controversy concerning the necessity of inguinal LN irradiation in the treatment of early stage node-negative ASCC. While many studies support elective inguinal LN irradiation^3,23–25^, some reports suggest its omission for early stage disease^26–31^. Further research is needed regarding elective inguinal LN irradiation in the treatment of early stage node-negative ASCC.

Consensus contouring guideline is essential to reduce inter/intra-clinician variability in the target volume delineation^32^. However, visualization of contouring guideline in the representative case has a limitation that it does not reflect the patients’ anatomic variations and diverse clinical scenarios. Research on the usefulness of auxiliary contouring tools such as Anatom-e (Anatom-e Information Systems Ltd., Houston, Texas), a digital platform facilitating target delineation by providing atlas as well as guidelines and protocols should also be considered^33^.

Several limitations exist in our study. We did not provide information on the exact radiotherapy treatment modality, dose prescription and PTV margin. Different institutional protocols may have affected clinicians' CTV delineation. Moreover, we did not investigate which guideline each clinician referred to mainly. Despite these limitations, this study addresses clinicians’ different consideration in target delineation of ASCC, which will help to establish a clearer target delineation guideline in the future.

In conclusion, moderate to substantial agreement was shown for ASCC CTV target delineation. However, large variations in the upper margin of the CTV-pelvis and the lower margin of the inguinal LN area suggest that further studies are needed to establish a clearer target delineation guideline.
